# Amide proton transfer imaging in differentiation of type II and type I endometrial carcinoma: a pilot study

**DOI:** 10.1007/s11604-021-01197-3

**Published:** 2021-09-15

**Authors:** Ryoya Ochiai, Naoko Mukuda, Hiroto Yunaga, Shinichiro Kitao, Kyohei Okuda, Shinya Sato, Tetsuro Oishi, Mitsuharu Miyoshi, Atsushi Nozaki, Shinya Fujii

**Affiliations:** 1 Division of Radiology, Department of Multidisciplinary Internal Medicine, Faculty of Medicine, Tottori University, 36-1, Nishi-cho, Yonago, Tottori 683-8504 Japan; 2 Department of Clinical Radiology, Tottori University Hospital, Yonago, Tottori 683-8504 Japan; 3 Division of Reproductive-Perinatal Medicine and Gynecologic Oncology, Department of Obstetrics and Gynecology, Faculty of Medicine, Tottori University, Yonago, Tottori 683-8504 Japan; 4 MR Applications & Workflow, GE Healthcare, 4-7-127 Asahigaoka Hino-shi, Tokyo, 191-8503 Japan

**Keywords:** Chemical exchange saturation transfer, Magnetic resonance imaging, Uterine, Differentiation, Histological

## Abstract

**Purpose:**

This study aimed at evaluating the efficacy of amide proton transfer (APT) imaging in differentiation of type II and type I uterine endometrial carcinoma.

**Materials and methods:**

Thirty-three patients diagnosed with uterine endometrial carcinoma, including 24 with type I and 9 with type II carcinomas, underwent APT imaging. Two readers evaluated the magnetization transfer ratio at 3.5 ppm [MTR_asym_ (3.5 ppm)] in each type of carcinoma. The average MTR_asym_ (APT_mean_) and the maximum MTR_asym_ (APT_max_) were analyzed. The receiver operating characteristic (ROC) curve analysis was performed.

**Results:**

The APT_max_ was significantly higher in type II carcinomas than in type I carcinomas (reader1, *p* = 0.004; reader 2, *p* = 0.014; respectively). However, APT_mean_ showed no significant difference between type I and II carcinomas. Based on the results reported by reader 1, the area under the curve (AUC) pertaining to the APT_max_ for distinguishing type I from type II carcinomas was 0.826, with a cut-off, sensitivity, and specificity of 9.90%, 66.7%, and 91.3%, respectively. Moreover, based on the results reported by reader 2, the AUC was 0.750, with a cut-off, sensitivity, and specificity of 9.80%, 62.5%, and 87.5%, respectively.

**Conclusion:**

APT imaging has the potential to determine the type of endometrial cancer.

## Introduction

Endometrial cancer is the most common malignancy of the female reproductive organs in developed countries, including the United States and Europe. The prevalence of the disease increases with the age of the population and the overall increase in the prevalence of obesity and metabolic syndromes in developed countries [1–4]. The prognosis of endometrial cancer is influenced by several factors, including the International Federation of Gynecology and Obstetrics (FIGO) staging system, vascular invasion, adnexal involvement, and lymph node metastasis [5, 6]. Most endometrial cancers are confined to the corpus uterus and have a good prognosis. However, the prognosis of these patients varies greatly, depending on three major factors: histopathological type, histological grade, and depth of myometrial invasion [5].

Bokhman [7] revealed that endometrial cancer is categorized into two types: type I and type II. Type I carcinoma is associated with estrogen hyperplasia, endometrial hyperplasia, frequent expression of estrogen and progesterone receptors, and younger age, whereas type II carcinoma, which is unrelated to estrogen, is associated with endometrial atrophy and older age [7–12]. Type I carcinoma, comprising well or moderately differentiated endometrial carcinoma, accounts for 80–90% of all endometrial carcinomas. Type II carcinoma, comprising histological types with a strong tendency to invade the myometrium, such as serous carcinoma and clear cell carcinoma, accounts for the remaining 10–20%. The classification of poorly differentiated endometrioid carcinoma as either type varies from report to report [8–12]. Although new classification methods based on the Cancer Genome Atlas (TCGA) currently have been investigated [8], the classification of endometrial cancer into type I and type II is clinically important to determine the treatment strategy. In fact, the National Comprehensive Cancer Network 2020 Guidelines [13] revealed that the combined use of pelvic and aortic lymphadenectomy may be considered in the management of type II carcinoma, owing to the high frequency of lymph node metastases. The guidelines also state that fertility preservation is not recommended, even though the cancer is confined to the uterus in type II carcinoma cases. Consequently, the preoperative differentiation of type II from type I carcinoma is very important to formulate a surgical treatment plan.

Amide proton transfer (APT) imaging is a novel imaging technique that uses endogenous contrast by exchange saturation transfer (CEST) to detect amide protons (–NH) in low-concentration solutes, such as mobile proteins and peptides in tissues or tumors [14]. Mobile proteins and peptides are thought to have a close relationship with tumor growth activity. The clinical utility of APT imaging has already been demonstrated in glioma, lung cancer, prostate cancer, and rectal cancers [15–18]. In gynecology, APT signals have demonstrated a correlation with the histologic grades of endometrioid endometrial carcinoma [19] and the efficacy in differentiating cervical cancer from normal cervical tissue [20].

The purpose of the current study was to characterize type II endometrial carcinoma using APT imaging and to evaluate the diagnostic accuracy of APT imaging in differentiating type II from type I carcinoma.

## Materials and methods

### Patients

The present study was approved by the Ethics Committee of our hospital, and all patients provided signed informed consent prior to scanning.

**Fig. 1:**
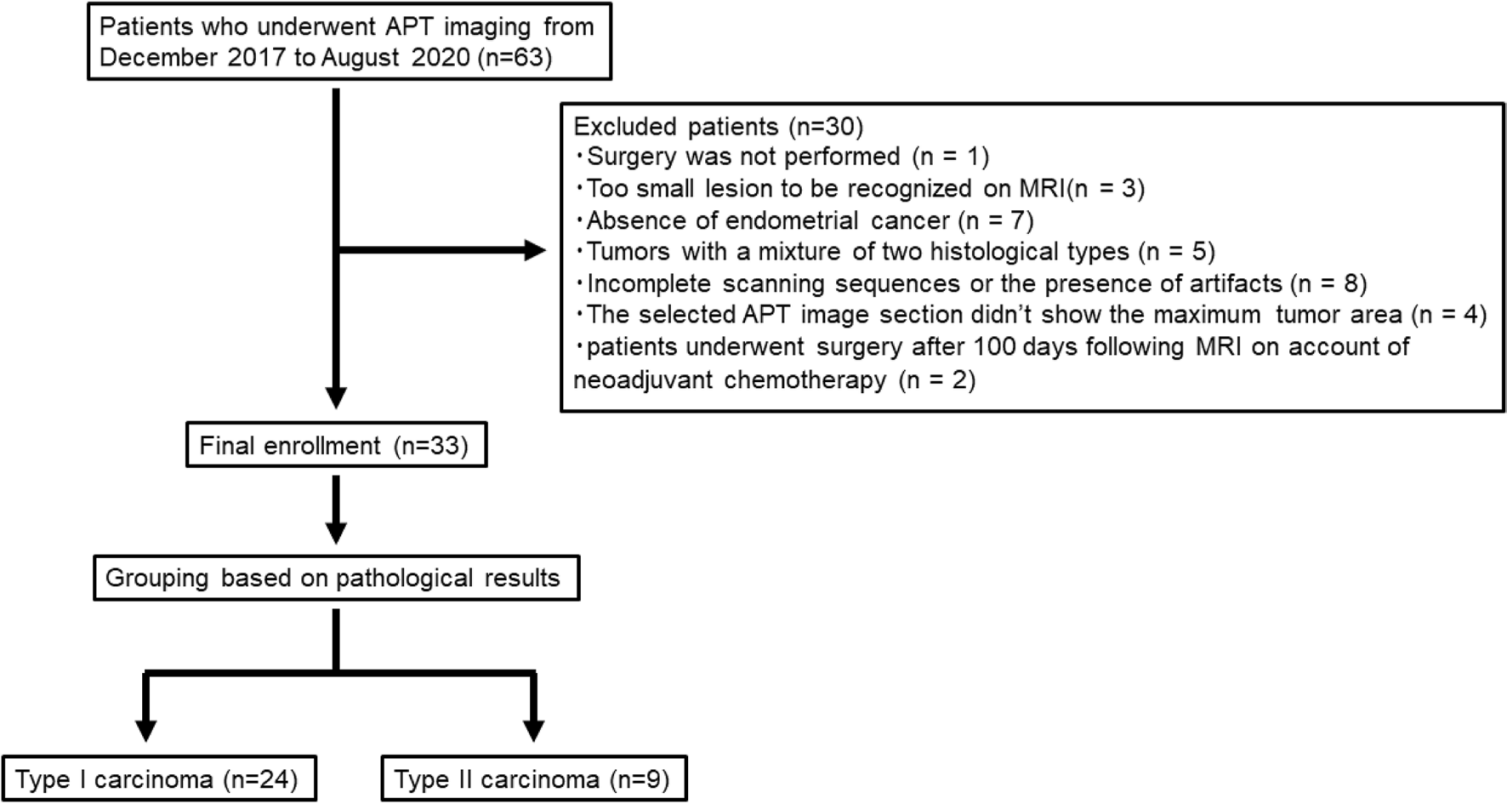
Flowchart depicting the patient selection

Between December 2017 and August 2020, 63 consecutive female patients underwent magnetic resonance imaging (MRI) because of the suspicion of endometrial cancer (Fig. 1). Among these patients, 30 were excluded for the following reasons: (1) surgery was not performed (*n* = 1); (2) lesions were too small to be recognized on MRI (*n* = 3); (3) absence of endometrial cancer (*n* = 7, two endometrial polyps, one atypical endometrial hyperplasia, one carcinosarcoma, one endometrial stromal sarcoma, and two patients with no significant tumor); (4) tumors with a mixture of two histological types (*n* = 5); (5) incomplete scanning sequences or the presence of too many motion/metal/air imaging artifacts (*n* = 8); (6) the selected APT image section did not show the maximum tumor area (*n* = 4); (7) patients underwent surgery after 100 days following MRI on account of neoadjuvant chemotherapy (*n* = 2). The final study population consisted of 33 patients (mean age 57.8 years; age range 37–72 years) with newly diagnosed endometrial carcinoma. All patients underwent surgery within 8–77 days after MR examination (mean time interval: 38 days). Macroscopic and microscopic pathological specimens were obtained during surgery.

On the basis of histological features, the well-differentiated (grade 1) and moderately (grade 2) differentiated endometrioid endometrial carcinomas were categorized as type I and poorly differentiated (grade 3) endometrioid endometrial carcinomas, serous carcinomas, and clear cell carcinomas were categorized as type II.

### MRI technique

MRI was performed using a 3 T MR system (Discovery MR750, GE Medical Systems, Milwaukee, Wisconsin, USA). A geometry embracing method anterior array 18 channel receiver array coil was used for signal reception. Before the examination, the patients were required to have full bladders. Conventional MRI parameters were as follows: sagittal T1-weighted imaging (T1WI) without fat suppression (field of view [FOV]: 25 × 25 cm^2^; slice thickness: 2.0 mm; spacing: 2.0 mm; number of slices: 82; repetition time [TR]: 4.6 ms; echo time [TE]: 2.2 ms; matrix: 512 × 512), sagittal T2-weighted imaging (T2WI) (field of view [FOV]: 25 × 25 cm^2^; slice thickness: 3.0 mm; spacing: 3.6 mm; number of slices: 31; repetition time [TR]: 6900 ms; echo time [TE]: 82 ms; matrix: 512 × 512), sagittal diffusion-weighted imaging (DWI) (field of view [FOV]: 24 × 24 cm^2^; slice thickness: 3.0 mm; spacing: 4.2 mm; number of slices: 23; repetition time [TR]: 4800 ms; echo time [TE]: 68 ms; matrix: 256 × 256; number of excitations: 6; *b* values: 0 and 800). After these sequences were acquired, 0.2 mL/kg of contrast agents (meglumine gadoterate or gadoteridol) were administered intravenously at a rate of 2.5 mL/s, followed by 30 mL of saline flush.

Two-dimensional sagittal APT imaging was performed using single-shot fast-spin echo (SSFSE) acquisition before the administration of contrast agents. The scanning parameters were as follows: TR: 5724 ms; echo time (TE): 31 ms; FOV: 30 × 24 cm^2^; matrix: 128 × 128; layer thickness: 5.0 mm; saturation pulse (RF): 1.7 μT; RF type: phase cycle; saturation time: 2500 ms; total acquisition time: 246 s. We used 43 images for these sequences, which comprised first idling, second image for S0 without saturation, third idling, and B0 correction images using water saturation shift referencing (± 240, ± 192, ± 144, ± 96, ± 48, 0 Hz), and NH images (± 896, ± 832, ± 768, ± 704, ± 640, ± 576, ± 512, ± 448, ± 384, ± 320, ± 256, ± 192, ± 128, ± 64, 0 Hz). The MTR_asym_ (3.5 ppm) was calculated at 3.5 ppm.

### Image analysis

The APT images were acquired through a single section selected by the radiological technicians as the tumor showing the maximum tumor area on sagittal T2WI and DWI. Subsequently, the MR data were analyzed using a clinical viewer (EV Insite; PSP, Tokyo, Japan). Images were independently analyzed by two radiologists (R.O. and N.M., with 3 and 8 years of experience in gynecological MRI, respectively) who were blinded to the histologic findings.

In each case, the radiologists drew a freehand region of interest (ROI) based on the lesion contour on the S0 image, which was obtained at the same scanning as that performed to capture the APT image. Each ROI was drawn by carefully reviewing the T2WI, DWI and contrast-enhanced images to determine the solid part of each tumor. Subsequently, the ROI was copied and pasted onto the MTR_asym_ image and apparent diffusion coefficient (ADC) map. We further adjusted the ROI after confirming whether the location of the ROI was appropriate. Tumor MTR_asym_ (3.5 ppm) and ADC values were determined. The ROI was placed to cover as much of the solid part of the tumor as possible and avoid large vessels, hemorrhage, calcification, cyst, necrosis, and normal myometrium. The average MTR_asym_ (APT_mean_), maximum MTR_asym_ (APT_max_), average ADC value (ADC_mean_), and minimum ADC value (ADC_min_) of each tumor were evaluated. The MTR_asym_ (3.5 ppm) can be calculated using the following equation [15]: MTR_asym_ (3.5 ppm) = (S_−3.5 ppm_ − S_3.5 ppm_)/S_0_; where S_−3.5 ppm_ and S_3.5 ppm_ are the signal intensities at − 3.5 and 3.5 ppm, respectively, and S_0_ is the signal intensity without saturation.

Additionally, reader 1 evaluated the distribution of the APT signal in the lesion, which was classified as either homogenous or heterogeneous. Heterogeneous distribution was further classified into three types based on the location of the high-signal APT spot—intratumoral focal high signal, high signal by necrosis, and high signal within myometrial invasion near tumor border. We defined high signal by necrosis as cases in which a high-signal APT spot was found around the non-enhancement area of the lesion. Therefore, the distribution of the APT signal was classified into four types.

### Statistical analysis

All statistical analyses were performed using SPSS 25.0. Values of *p* < 0.05 were considered to be significant. The Shapiro–Wilk test was used to evaluate the normality of distribution of data, which did not show a normal distribution. Subsequently, the Mann–Whitney *U* test was used to evaluate the differences between type I and type II carcinomas with regard to the APT_mean_ and APT_max_. Additionally, we evaluated the differences with regard to the ADC_mean_ and ADC_min_. We also evaluated the correlation between APT_mean_ and ADC_mean_ as well as APT_max_ and ADC_min_ using Spearman’s rank correlation coefficient.

The interobserver agreement for the ROI measurements pertaining to the two readers was analyzed by estimating the interclass correlation coefficient (ICC 0.00–0.20 slight, 0.21–0.40 fair, 0.41–0.60 moderate, 0.61–0.80 substantial, and 0.81–1.00 almost perfect correlation).

ROC curve analysis was used to evaluate the diagnostic accuracy of APT_mean_ and APT_max_. Additionally, by maximizing the Youden index (defined as sensitivity + specificity − 1), the optimal thresholds and corresponding sensitivities and specificities for the differentiation of type II and type I carcinomas were determined.

## Results

### Patient characteristics

Histopathological evaluation of the 33 postoperative specimens revealed 24 type I and nine type II endometrial carcinomas. Among the 24 type I endometrial carcinomas, 19 were grade 1, and five were grade 2 endometrioid carcinomas. Among the nine type II endometrial carcinomas, four were grade 3 endometrioid carcinomas, three were serous carcinomas, and two were clear cell carcinomas. The patient characteristics are summarized in Table 1.

**Table 1:** Patient characteristics

	Type I	Type II	*p* value
Mean age	56.1 ± 12.1	61.1 ± 4.28	0.090
Tumor size (mm)	49.7 ± 26.3	73.6 ± 32.8	0.037
Histological type			
Endometrioid			
Grade 1	19		
Grade 2	5		
Grade 3		4	
Serous		3	
Clear cell		2	
FIGO staging			
IA	17	3	
IB	5	2	
II	1		
IIIA	1		
IIIC2		1	
IVB		3	

### APT signals and ADC values in type I and II endometrial carcinoma

**Fig. 2:**
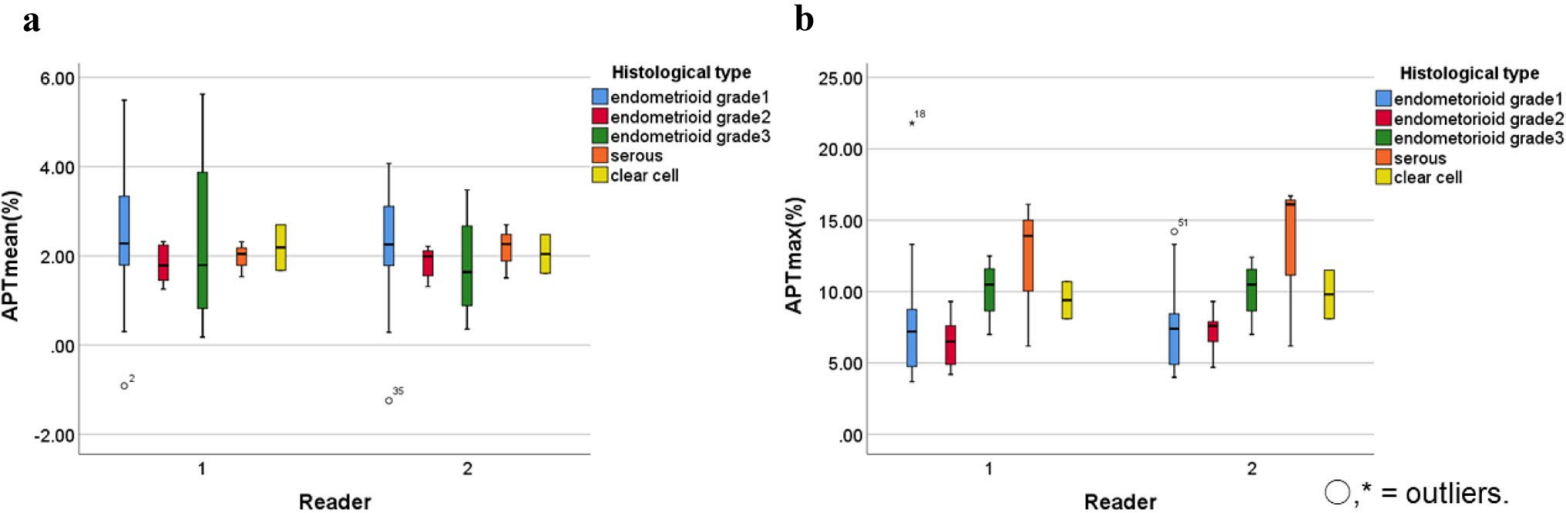
Box-and-whisker plots show the distribution of the APT_mean_ (**a**) and the APT_max_ (**b**) in each histopathological classification of carcinomas. Boxes represent values from the lower to upper quartiles. The central line represents the median, and small circles and asterisks represent the extreme values (outliers). Whiskers extend from the minimum to maximum values, excluding the outliers

**Fig. 3:**
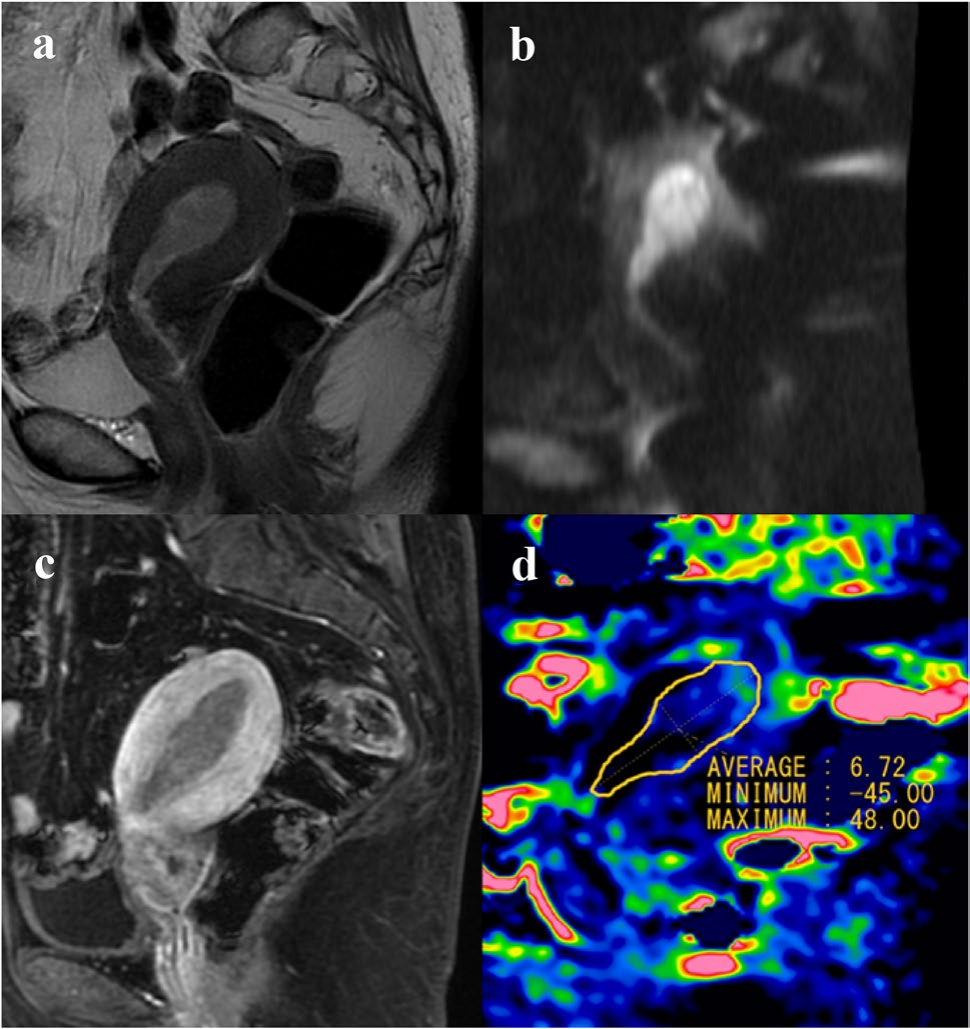
Images and placement of the ROI in a case of grade 1 endometrioid endometrial carcinoma. **a** Represents the T2WI, **b** represents the DWI (*b* = 800), and **c** represents the contrast enhanced T1WI, **d** represents MTR_asym_ (3.5 ppm) image. The values shown in the figure are multiplied by 1000 to obtain an integer value. Consequently, a value of 10 in the figure is 1.0%. The MTR_asym_ (3.5 ppm) image does not show a very high signal area within the ROI. The APT_mean_ and APT_max_ obtained by reader 1 were 1.89% and 4.90%, respectively. The APT_mean_ and APT_max_ obtained by reader 2 were 0.67% and 4.80%, respectively. The APT signal pattern was classified as homogenous

**Fig. 4:**
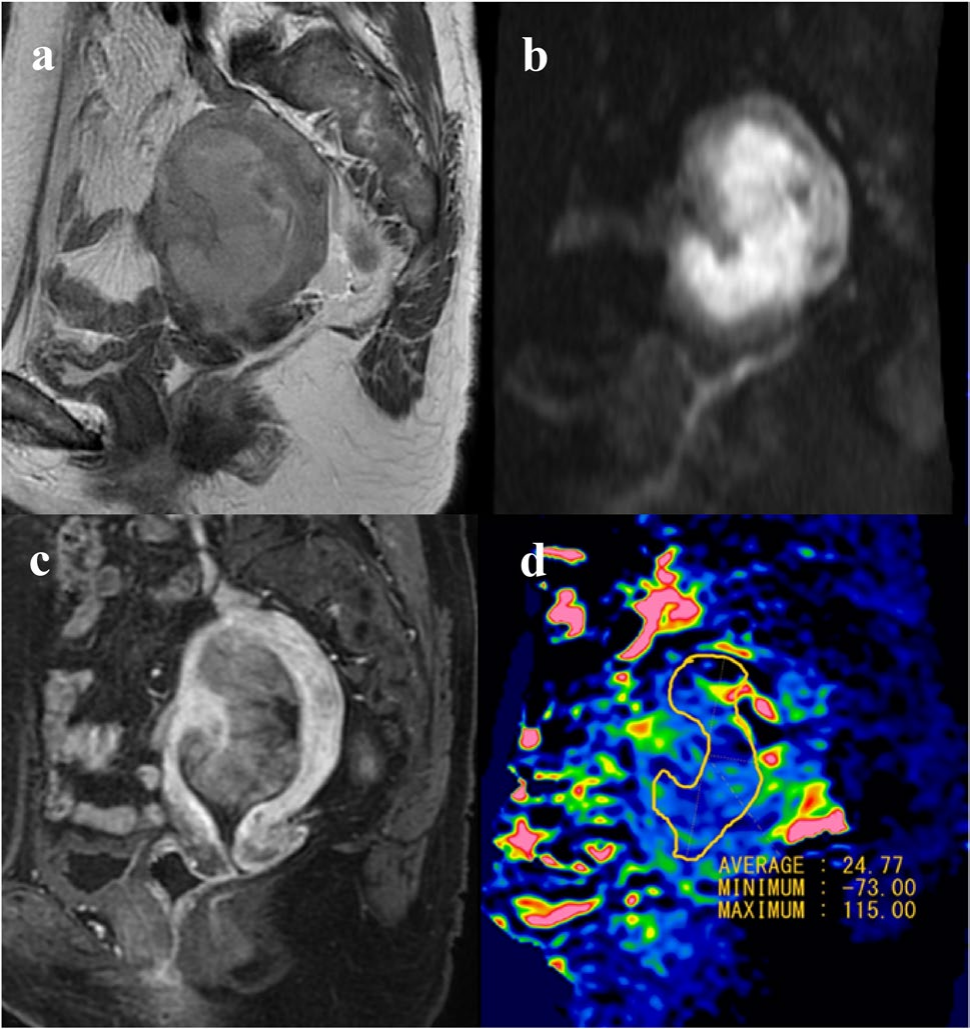
Images and placement of the ROI in a case of clear cell carcinoma. **a** Represents the T2WI, **b** represents the DWI (*b* = 800), and **c** represents the contrast enhanced T1WI, **d** represents MTR_asym_ (3.5 ppm) image. The values shown in the figure are multiplied by 1000 to obtain an integer value. Consequently, a value of 10 in the figure is 1.0%. The MTR_asym_ (3.5 ppm) image shows very high signal areas within the ROI. The APT_mean_ and APT_max_ obtained by reader 1 were 2.78% and 12.7%, respectively. The APT_mean_ and APT_max_ obtained by reader 2 were 2.48% and 11.50%, respectively. The APT signal pattern was classified as high signal by necrosis

The ICCs pertaining to APT_mean_ and APT_max_ were 0.86 and 0.90, respectively, indicating an almost perfect correlation. The ROI area was 7.92 ± 7.47 cm^2^ for reader 1 and 8.28 ± 9.37 cm^2^ for reader 2. A summary of the results of the two readers is shown in Tables 2 and 3. The APT_max_ was significantly higher in type II carcinomas than in type I carcinomas (reader 1: *p* = 0.004; reader 2: *p* = 0.014) (Fig. 2). Conversely, there was no significant difference between type I and type II carcinomas with regard to APT_mean_ (reader 1: *p* = 0.648; reader 2: *p* = 0.619) (Fig. 2). Figures 3 and 4 show representative images. The APT signal patterns for each histopathological classification are shown in Table 4.

**Table 2:** Comparison between type I and type II carcinomas with regard to the APT and ADC

	Reader 1			Reader 2		
	Type I	Type II	*p*	Type I	Type II	*p*
APT_mean_	2.21 ± 0.93	2.18 ± 1.47	0.648	2.26 ± 0.98	1.96 ± 0.89	0.619
APT_max_	6.64 ± 2.46	10.61 ± 3.23	0.004	7.05 ± 2.37	11.00 ± 3.69	0.014
ADC_mean_	1.04 ± 0.20	1.06 ± 0.18	0.512	1.00 ± 0.22	1.03 ± 0.19	0.858
ADC_min_	0.60 ± 0.17	0.48 ± 0.19	0.183	0.62 ± 0.15	0.52 ± 0.18	0.246

*APT* amide proton transfer, *ADC* apparent diffusion coefficient

**Table 3:** APT_mean_ and APT_max_ for each histopathological classification

	Endometrioid				
	Grade 1	Grade 2	Grade 3	Serous	Clear cell
Reader 1					
APT_mean_	2.32 ± 1.01	1.81 ± 0.47	2.35 ± 2.33	1.97 ± 0.40	2.19 ± 0.72
APT_max_	6.68 ± 2.62	6.50 ± 2.06	10.1 ± 2.29	12.1 ± 5.20	9.40 ± 1.84
Reader 2					
APT_mean_	2.38 ± 1.07	1.83 ± 0.39	1.78 ± 1.30	2.16 ± 0.60	2.04 ± 0.61
APT_max_	7.01 ± 2.58	7.20 ± 1.72	10.1 ± 2.26	13.0 ± 5.90	9.80 ± 2.40

*APT* amide proton transfer

**Table 4:** APT signal patterns for each histopathological classification

	Endometrioid				
	Grade 1	Grade 2	Grade 3	Serous	Clear cell
Homogenous	9	1			
Heterogenous					
Intratumoral focal high signal	4	1		1	
High signal by necrosis	2	2	2	1	2
High signal within myometrial invasion near tumor border	4	1	2	1	

We further performed a sub-analysis of only endometrioid carcinomas. The APT_max_ was significantly higher in type II (grade 3) endometrioid carcinomas than in type I (grade 1 and 2) endometrioid carcinomas with regard to the results of reader 1 (reader 1: *p* = 0.034; reader 2: *p* = 0.070). There was no significant difference between type I and type II endometrioid carcinomas with regard to APT_mean_ (reader 1: *p* = 0.728; reader 2: *p* = 0.336).

ADC_mean_ and ADC_min_ were not significantly different between type I and type II carcinomas (ADC_mean_; reader 1, *p* = 0.512; reader 2, *p* = 0.858; ADC_min_, reader 1: *p* = 0.183; reader 2: *p* = 0.246).

### Correlation between APT signals and ADC values

We found no significant correlation between APT_mean_ and ADC_mean_ (reader 1: *p* = 0.200, reader 2: *p* = 0.610) or between APT_max_ and ADC_min_ (reader 1: *p* = 0.258; reader 2: *p* = 0.974).

### ROC analysis

**Fig. 5:**
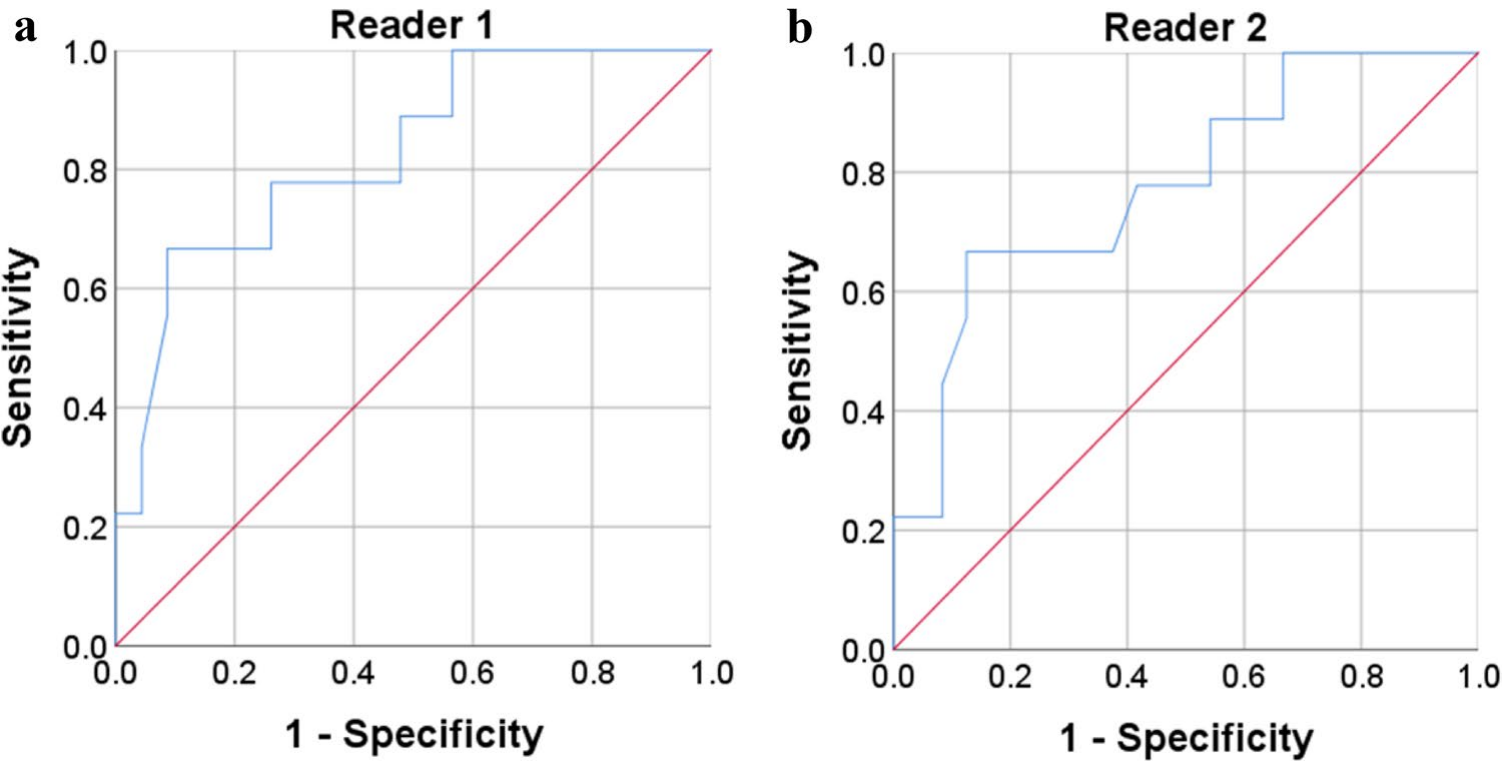
Receiver operating characteristic (ROC) curves show the results of the analysis of APT_max_ obtained by reader 1 (**a**) and reader 2 (**b**). Details of area under the curves (AUC) and 95% CIs of each index are shown in the “Results” section

On the basis of the results reported by reader 1, the AUC pertaining to the APT_max_ for distinguishing type I from type II carcinomas was 0.826 (95% confidence interval [CI] 0.667–0.985), with a cut-off, sensitivity, and specificity of 9.90%, 66.7%, and 91.3%, respectively. Moreover, on the basis of the results reported by reader 2, the AUC pertaining to the APT_max_ for distinguishing type I from type II carcinomas was 0.750 (95% CI 0.556–0.944), with a cut-off, sensitivity, and specificity of 9.80%, 62.5%, and 87.5%, respectively (Fig. 5).

## Discussion

The present study demonstrated that type II endometrial carcinoma has a higher APT_max_ than type I endometrial carcinoma, although there were no significant differences between type I and type II carcinomas with regard to the APT_mean_. Related reports suggest that the tumor APT signal is positively correlated with cell density and proliferative capacity. Furthermore, this is attributed to an increase in the concentration of mobile proteins and peptides in the cell [15, 19, 21]. Hence, high cellularity contributes to increased APT signal. Nuclear atypia is another possible factor related to increased APT signals, owing to interactions with hydrophobic cell membranes and macromolecules [22–24]. Serous and clear cell carcinomas also exhibited higher APT_max_ signals. They are suggested to be marked nuclear atypia, prominent mitoses, and proliferation of solid components [8, 9]. Endometrioid carcinoma is classified into architectural grades (grades 1–3), in accordance with the percentage of solid part as defined by FIGO; grades 1 and 2 contain less than 50% solid part and are predominantly glandular, whereas grade 3 contains more than 50% solid part as well as marked nuclear atypia [9, 19]. These differences, including cellularity and nuclear atypia, may contribute to the abundant concentration of mobile proteins and peptides presented by type II carcinomas, namely the higher APT_max_ signal. Nevertheless, in the present study, the APT signal was not correlated with the ADC value, reflecting tumor cellularity. Similarly, APT_mean_ did not show a certain increase according to the tumor grade in endometrioid carcinoma. These results may be due to the small number of cases investigated. Moreover, other factors, in addition to nuclear atypia and prominent mitoses, may have contributed to the APT signal much more.

In general, APT_max_ can indicate the area with the highest local cell density or the most active metabolic activity. High APT signal spots were found in the border area between the tumor and the myometrium, particularly in type II carcinoma. According to a recent study by Gatius et al. [25], the proteomic and metabolic features differ between peripheral and inner cells in tumors with myometrial invasion. In general, type II carcinomas tend to be invasive in nature compared to type I carcinomas. This difference may produce high APT signal spots in type II carcinomas, which reflect active metabolism at the intra-myometrial tumor front. Furthermore, necrosis can increase the APT signal [15, 20]. Although we drew the ROI excluding necrosis, microscopic necrosis could exist in the ROI. Additionally, morphological differences, including cytological (columnar, mucinous, or squamous) or architectural (glandular, papillary, or solid) features, are frequently observed in the same tumor [25]. Thus, we believe that tumor heterogeneity can affect the results. Therefore, APT may be a promising imaging marker that provides a good representation of not only the histopathological characteristics but also tumor heterogeneity related to the microenvironmental metabolic characteristics of the tumor.

The present study included grade 3 endometrioid carcinoma as type II, and it is debatable whether grade 3 endometrioid carcinoma is type I or type II. However, Voss et al. [26] reported that grade 3 endometrioid carcinoma may be more suitable for inclusion in the category of type II, as the immunohistochemistry and survival profiles are similar to those of serous and clear cell carcinomas. Moreover, the conventional classification of endometrial carcinoma into type I and type II has been recently questioned, owing to the frequent disagreement between pathologists with regard to the diagnosis due to the similarity of the histological characteristics pertaining to grade 3 endometrioid and serous carcinomas [8–11].

Previous studies have also attempted to differentiate type II carcinoma from type I carcinoma using MRI. Regarding ADC values, a recent study showed that the mean and minimum ADC values were significantly lower in type II carcinoma than in type I carcinoma [27], although we did not obtain the same results. Differentiation was assessed using dynamic contrast-enhanced MRI [12] and MR spectroscopy [28]. However, these evaluations are debatable. APT_max_ can show the tumor microenvironment, as mentioned above. Therefore, it may provide more detailed information that would not be available with conventional evaluation methods.

The current study has several limitations. First, the number of cases was relatively small, especially in cases of type II carcinoma. Second, APT imaging was performed for only one section per patient, owing to the time limitations of the imaging protocol. To visualize all areas of a tumor, multiple scans, such as the 3D protocol, are suitable and should be employed in future studies involving the same. Third, we excluded four cases because of inappropriate slice selection. APT imaging is performed before the administration of contrast media, as contrast media affects CEST contrast [29]. In these cases, we were not able to obtain the image with the largest tumor area because the post-contrast enhanced images showed the outline of the tumor more clearly than pre-contrast enhanced images. Finally, we believe that we cannot completely exclude the impact of artifacts, such as those caused by intestinal motion, on the APT signal, in relatively small lesions, although APT imaging was performed using the SSFSE-based sequence. In particular, the relatively long examination time for APT imaging is a major problem as artifacts caused by intestinal motion are strongly affected. To solve this problem, we have to shorten the TR using various methods. For example, using a continuous wave instead of a phase cycle as the RF type would shorten the saturation time or reduce the number of refocus RF pulses of SSFSE.

In conclusion, APT_max_ was observed to be higher in type II than in type I endometrial carcinoma. APT imaging has the potential to determine the type of endometrial cancer, which can facilitate preoperative decision making.
